# Supplementary material for: Model to Predict Healthcare Behaviors: Comparison of a Chilean and Mexican Sample
Source: Int J Environ Res Public Health. 2022 Aug 15;19(16):10067. doi: 10.3390/ijerph191610067 (PMC9407967; doi:10.3390/ijerph191610067)
Supplement: Supplementary file 1 [file ijerph-19-10067-s001.zip › ijerph-1775409-supplementary.pdf]

International Journal of
Environmental Research
and Public Health

MDPI

Supplementary Material

Table S1. Comparison between the variables by sex.

Variable	Men (SD)	Women (SD)	*p*	*t*
Negative beliefs about physicians	2.81 (0.69)	2.78 (0.72)	0.61	0.50
Perception of mistreatment in health	1.85 (0.62)	1.76 (0.60)	0.11	1.60
Anger	2.52 (1.25)	2.65 (1.42)	0.32	0.97
Anxiety	2.13 (1.06)	2.16 (1.15)	0.72	0.35
Sadness	2.22 (1.17)	2.31 (1.25)	0.46	0.73
Demotivation	2.94 (1.14)	3.03 (1.18)	0.40	0.84
Delay medical care	2.74 (1.06)	2.83 (1.12)	0.38	0.88
Refuse help of healthcare providers	2.87 (1.06)	3.02 (1.11)	0.17	1.37
